# Combination therapy of ginsenoside compound K and methotrexate was efficient in elimination of anaemia and reduction of disease activity in adjuvant-induced arthritis rats

**DOI:** 10.1080/13880209.2020.1844761

**Published:** 2020-11-16

**Authors:** Jingyu Chen, Wu Wang, Mengya Jiang, Mei Yang, Wei Wei

**Affiliations:** Key Laboratory of Anti-inflammatory and Immune Medicine, Institute of Clinical Pharmacology, Anhui Collaborative Innovation Center of Anti-inflammatory and Immune Medicine, Anhui Medical University, Ministry of Education, Hefei, China

**Keywords:** Rheumatoid arthritis, anaemia of inflammation, natural products of ginsenosides

## Abstract

**Context:**

Ginsenoside compound K (CK) has anti-inflammatory, immunoregulatory, and myelosuppressive protective effects. Methotrexate (MTX) is widely used in combination therapy for rheumatoid arthritis (RA).

**Objective:**

To evaluate the effects of combination therapy of CK and MTX on anaemia and anti-arthritis in adjuvant-induced arthritis (AA) rats.

**Materials and methods:**

AA was induced in rats by Complete Freund’s adjuvant, and divided into five groups (*n* = 10): normal, AA, CK 80 mg/kg, combination therapy (80 mg/kg CK combined with 0.5 mg/kg MTX), and MTX 0.5 mg/kg. From day 12, CK (once a day for 15 days) or MTX (once every 3 days, five times) were intragastrically administered.

**Results:**

Combination therapy showed increased haemoglobin to 148.5 ± 10.1 g/L compared with AA (129.8 ± 11.7 g/L) and MTX (128.8 ± 18.4 g/L), and decreased reticulocytes in peripheral blood to 4.9 ± 1.1% compared with MTX (9.3 ± 3.3%). In combination therapy group, paw swelling decreased to 5.6 ± 4.3 mL compared with CK (9.4 ± 3.9 mL) and MTX (13.5 ± 7.4 mL), and swollen joint count decreased to 1.4 ± 0.8 compared with CK (2.1 ± 1.0) and MTX (2.4 ± 1.2) at day 24. Combination therapy showed decreased IL-6 to 25.1 ± 17.2 pg/mL compared with MTX (44.9 ± 4.8 pg/mL), and decreased IL-17 to 5.8 ± 3.9 pg/mL compared with MTX (10.7 ± 4.2 pg/mL).

**Conclusion:**

The anti-anaemia effect of CK deserves further study, and CK can be a candidate effective drug for combined treatment in RA with anaemia.

## Introduction

Rheumatoid arthritis (RA) is a systemic autoimmune disease, and the main manifestations are chronic synovitis, bone erosion, joint dysfunction and other extra-articular symptoms. Anaemia is the common extra-articular symptom of RA, which appears in about 30.4% male and 32.0% female RA patients (Wolfe and Michaud 2006; Ganna 2014). Anaemia is the independent factor of body dysfunction in RA patients. After effective treatment, with the improvement of anaemia, the body function will also be improved (Han et al. 2007). Because the pathogenesis of RA is still unclear, there is no satisfactory clinical treatment. Although methotrexate (MTX) is still recommended as the basis drug for RA treatment. Low-dose MTX works slowly and usually causes many adverse reactions after long-term use such as hematological toxicity including anaemia (Gilani et al. 2012; Dubey et al. 2016). To avoid adverse reactions and achieve better efficiency, MTX is widely used in combination therapy for RA, such as MTX in combination with a monoclonal antibody, a chemical compound, or natural extraction products (Colebatch et al. 2011; Mori et al. 2019). However, few methods have specific effect on anaemia in RA. It is meaningful to search for a rational combination therapy in the treatment of RA, which can achieve better efficacy and eliminate anaemia at the same time.

Ginseng has been used as medicine and health food for a long time. A large number of studies have found that ginsenosides play a pharmacological role in immune regulation, anti-inflammation, antioxidation and antitumor (Zeng et al. 2018; Jung et al. 2019; Zhang et al. 2018). Ginsenoside compound K (CK) comes from the degradation of products of natural diol-type ginsenosides (Rb1, Rb2 and Rc) in the human intestinal tract, and is the main form of ginsenoside absorption and activity *in vivo*. Recently, a study showed that CK protected bone marrow from cyclophosphamide by controlling cell apoptosis and cell cycle (Han et al. 2019). Our previous studies showed that CK exerted definite effect in the treatment of collagen-induced arthritis (CIA) mice (Liu et al. 2014; Chen et al. 2015; Zhang et al. 2019) and adjuvant-induced arthritis (AA) rats (Chen et al. 2014, 2016; Wu et al. 2014). In this study, the advantages of anti-arthritis effects and the benefits on anaemia were evaluated using a combination of CK and MTX.

## Materials and methods

### Animals

Fifty Sprague Dawley (SD) rats, male, weighing 150 ± 20 g, were purchased from Anhui Laboratory Animal Centre, licence number SCXK (Anhui) 2015-002. The experimental schemes involved in the experiment were approved by the Animal Ethics Committee of the Institute of Clinical Pharmacology, Anhui Medical University.

### Reagents

CK (No: S141001) was transformed from ginsenosides by microbial fermentation technology in Zhejiang Haizheng Pharmaceutical Co., Ltd (Taizhou, China). MTX (No.036140202) was purchased from Shanghai Xinyi Pharmaceutical Factory Co., Ltd (Shanghai, China). Rats were given CK or MTX with 0.5% sodium carboxymethylcellulose suspension, intragastric administration. BCG vaccine (20140804) was purchased in Chengdu Biological Products Co., Ltd (China). Lipopolysaccharides (LPS) and ConA were products of Sigma (USA). ELISA kits were purchased from ebioscience (USA), Raybiotech (USA) and CUSABIO (China), including TNF-α, IFN-γ, IL-1β, IL-2, IL-6, IL-17, PGE2, osteoprotegerin (OPG) and receptor activator of nuclear factor-κB ligand (RANKL). The Cell Counting Kit-8 was a product of Dojindo Molecular Technologies, Inc. (Tokyo, Japan).

### AA induction and treatment

Complete Freund’s adjuvant (10 mg/mL) was injected intracutaneously into the right hind paw of rats to induce inflammation. Saline was injected into rats in normal group in the same way. The day of injection was regard as day 0. These AA rats were randomly divided into 4 groups (*n* = 10) including AA, CK 80 mg/kg, combination therapy group (80 mg/kg CK combined with 0.5 mg/kg MTX), and MTX 0.5 mg/kg. From the 12th day (the onset of arthritis), CK (once a day for 15 days) or MTX (once every 3 days) were intragastrically administered. Rats in normal group and AA group were given the same amount of sodium carboxymethyl cellulose solution.

### Assessment of AA

To evaluate the severity of arthritis, global assessment, arthritis index, swollen joint count, and paw volume were evaluated. From the day 12, two observers who were blinded to the groups evaluated these indicators. Global assessment was based on symptoms in different parts of rats, including ear, nose, tail, and paws (Chen et al. 2018). Each rat paw has five phalanx joints and one ankle or wrist joint; the number of swelling joints was counted. Degree of joint swelling was recorded and scored as arthritis index (Wu et al. 2007). The secondary inflammatory paw (left hind) swelling of rats was evaluated at day 0, 12, 15,18, 21, 24, 27 using a Paw Volume Metre YLS-7A (Equipment station of Academy of Medical Sciences, Shandong, China). Paw volume (△mL)=paw volume (day 12, 15,18, 21, 24, 27) – paw volume (day 0).

### Erythrocyte and haemoglobin

Peripheral blood was collected from rats on day 27, and then the following items were tested: the number of erythrocytes, haemoglobin concentration, the number and proportion of reticulocytes.

### Histological examination

Rats were killed at day 27, following anesthesia. The hind limbs and spleen were embedded in paraffin after fixed with 10% neutral formalin. Paraffin sections (5 μm) were made and stained with haematoxylin and eosin, and were examined by microscope. Histopathology of the joints and spleen was assessed by observers who were blinded to the groups. Synovial hyperplasia, cell infiltration in synovium, pannus formation, and cartilage erosion in joints were assessed (Wu et al. 2016). The periarteriolar lymphosheath, lymphoid follicles, and marginal areas in spleen were evaluated (Chen et al. 2018).

### Preparation of macrophages

Peritoneal lavage cells were collected aseptically and washed twice with Hanks solution. After cell counting, 100 μL of cells (2 × 10^6^/mL) were inoculated into a 24-well cell culture plate. The cells were cultured at 37 °C and 5% CO_2_ incubator under complete humidity for 2 ∼ 4 h. The cells without adherence were washed twice with Hanks solution and the macrophages were obtained.

### Preparation of splenic mononuclear cells

Monocytes were obtained from the single cell suspension of the spleen by gradient centrifugation. After washing with PBS for three times, the concentration of monocytes was adjusted to 5 × 10^6^ cells/mL. Then these cells were used for the activity determination of T cells and B cells.

### Preparation of fibroblast like synoviocytes (FLS)

Rats were immersed and disinfected in 0.1% chlorhexidine after sacrificed, and the synovial tissue was aseptically taken, cut into small pieces of 1 ∼ 2 mm^2^, and arranged in a culture flask to adhere to the wall. The culture medium was changed every 2–3 days. When a large number of fibroblasts grew out, the tissues was gently removed until the cells were almost confluent. Then cells were digested with 0.25% trypsin and subcultured, and the third-generation cells were used for experiments.

### Detection of cell viability

Viability of T, B and FLS was assayed by Cell Counting Kit. Spleen mononuclear cells (200 μL, 1 × 10^6^) were co-cultured with ConA (4 mg/mL) or LPS (5 mg/mL) respectively in 96-well plates in a incubator with 5% CO_2_ at 37 °C for 24 h. FLS were co-cultured with TNF-α (20 ng/mL) in 96-well plates in an incubator with 5% CO_2_ at 37 °C for 24 h. Cells were cultured at 37 °C for an another 2 h after CCK-8 reagent (10 μL per well) was added. Then the absorbance at 450 nm was measured by a microplate reader.

### Cytokine detection

Cytokines in serum and secreted by FLS and macrophages were detected by ELISA. FLS or macrophages (1 × 10^5^ cells/mL) was added to 24-well plates (500 μL per well), and cultured in an incubator at 37 °C with 5% CO_2_ for 48 h. The supernatant was collected and the cytokine levels were measured by ELISA. Serum was collected from rats on day 27, and cytokines were detected by ELISA.

### Statistical analysis

Data were represented by mean ± SD. Differences among multiple groups were conducted with one-way analysis of variance, and *p* values <0.05 were considered to be significant.

## Results

### Combined effects of CK and MTX on arthritis in AA rats

**Figure 1.**
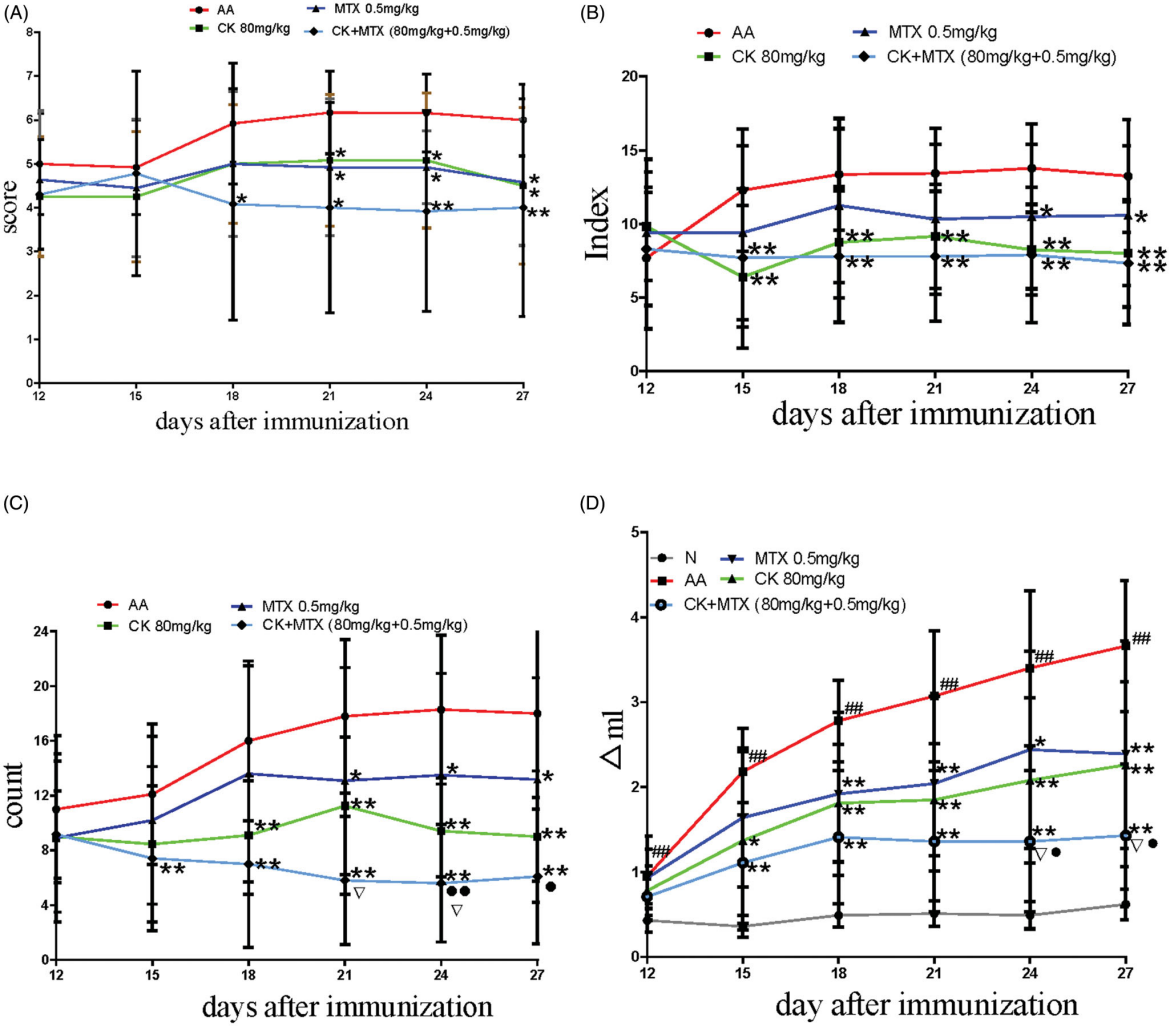
Combined effects of CK and MTX on arthritis signs in AA rats. (A) global assessment; (B) arthritis index; (C) swollen joint counts; (D) paw swelling; Data are expressed as the mean ± SD, with 10 animals in each group. ^##^*p* < 0.01 vs. normal; *p < 0.05; **p < 0.01 vs. AA; ^▽^*p* < 0.05 vs. MTX; ^●^*p* < 0.05; ^●●^*p* < 0.01 vs. CK.

To evaluate the therapeutic effect of combination therapy on AA rats, combination of CK and MTX were administered to AA rats from day 12 to day 27. The results revealed that combination of CK and MTX reduced the global assessment, arthritis index, swollen joint count and paw swelling. Combination of CK and MTX was superior to CK alone or MTX alone in reduction the paw swelling and swollen joint count. In combination therapy group, paw swelling decreased to 5.6 ± 4.3 mL compared with CK (9.4 ± 3.9 mL) and MTX (13.5 ± 7.4 mL) at day 24, and swollen joint count decreased to 1.4 ± 0.8 compared with CK (2.1 ± 1.0) and MTX (2.4 ± 1.2) at day 24 (Figure 1).

### Combined effects of CK and MTX on joint erosion and synovium inflammation in AA rats

**Figure 2.**
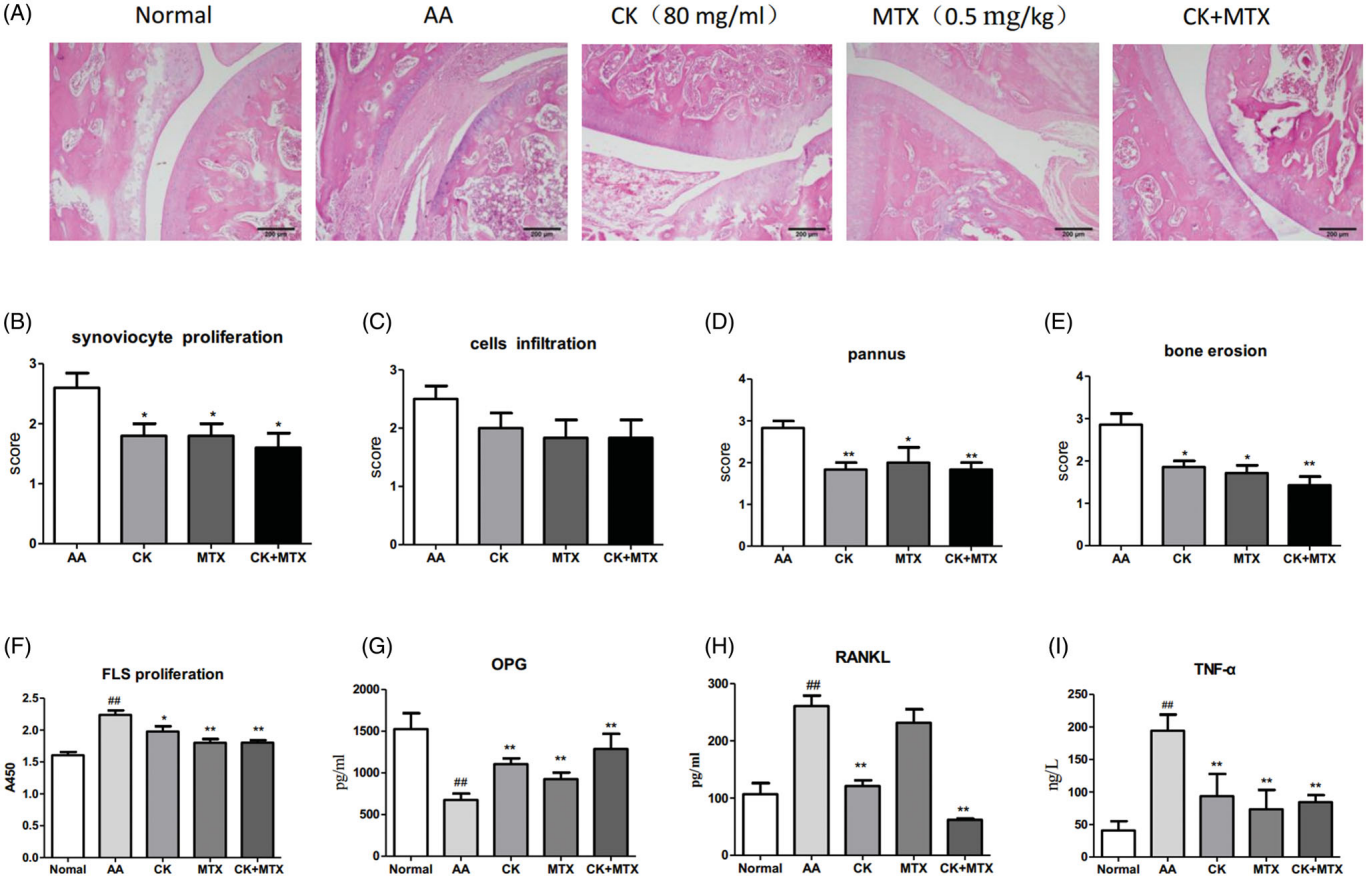
Combined effects of CK and MTX on joint erosion and FLS function in AA rats. (A) Photomicrographs of the rat joint (original magnification × 100, haematoxylin and eosin stain). (B) synoviocyte proliferation; (C) inflammatory cellular infiltration; (D) pannus formation; (E) bone erosion. (F) FLS proliferation; (G) the secretion of OPG from FLS; (H) the secretion of RANKL from FLS; (I) the secretion of TNF-α from FLS. Data are expressed as the mean ± SD. ^##^*p* < 0.01 vs. Normal; *p < 0.05; **p < 0.01 vs. AA. Data are expressed as the mean ± SD, with 6 animals in each group.

Combination of CK and MTX improved FLS proliferation, inflammatory cell infiltration, pannus formation and bone erosion in joint of AA rats (Figure 2(A–E)). The proliferation of FLS from AA rats was evidently increased. Compared with AA rats, combination of CK and MTX could significantly down-regulate the proliferation ability of FLS (Figure 2(F)). RANKL and OPG, which are secreted mainly by synovial cells, contribute to bone and joint erosion in RA. Compared with FLS from normal rats, the secretion of OPG in FLS from AA rats reduced, and the secretion of RANKL and TNF-α increased significantly. Compared with AA FLS, TNF-α and RANKL levels were reduced, and secretion of OPG were increased in the combination therapy group (Figure 2(G–I)).

### Combined effects of CK and MTX on erythrocyte and haemoglobinin AA rats

**Figure 3.**
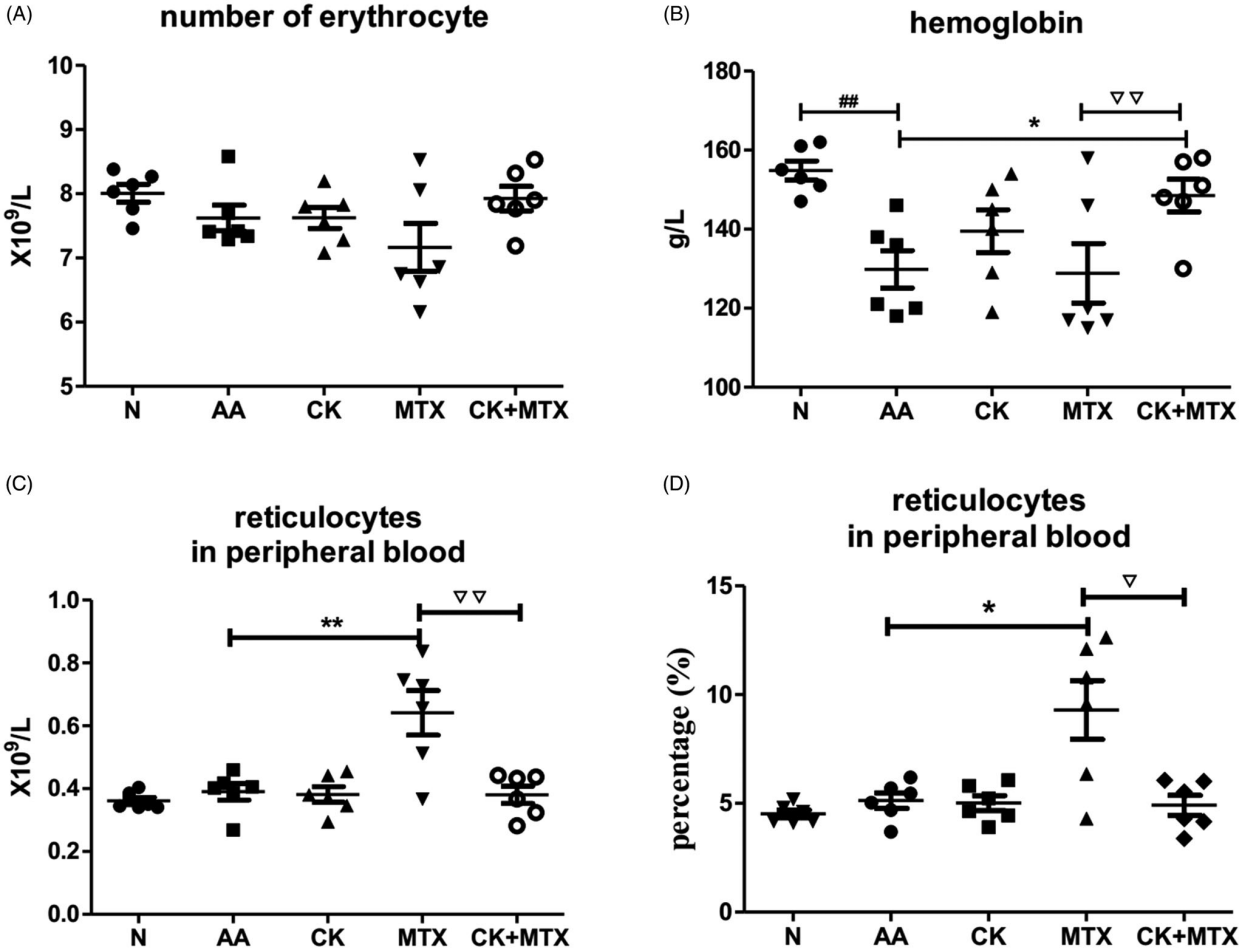
Combined effects of CK and MTX on erythrocyte and haemoglobin in AA rats. (A) number of peripheral blood erythrocyte; (B) haemoglobin concentration in peripheral blood; (C) number of reticulocytes in peripheral blood; (D) proportion of reticulocytes in peripheral blood. Data are expressed as the mean ± SD, with 6 animals in each group. ^##^*p*＜0.01 vs. Normal; *p＜0.05; **p＜0.01 vs. AA; ^▽^*p*＜0.05; ^▽▽^*p*＜0.05 vs. MTX.

Although the number of erythrocyte and reticulocytes in peripheral blood were unchanged in AA rats (Figure 3(A,C,D)), the haemoglobin concentration in peripheral blood of AA rats was reduced (*p* < 0.01) when compared with rats in normal groups. Combination therapy showed increased haemoglobin concentration to 148.5 ± 10.1 g/L compared with AA group (129.8 ± 11.7 g/L) and MTX group (128.8 ± 18.4 g/L), (Figure 3(B)). Administration of MTX significantly increased the number and proportion of reticulocytes in peripheral blood of AA rats, while combination therapy showed a decreased proportion of reticulocytes in peripheral blood to 4.9 ± 1.1% compared with MTX group 9.3 ± 3.3% (Figure 3(C,D)).

### Combined effects of CK and MTX on immune function in AA rats

**Figure 4.**
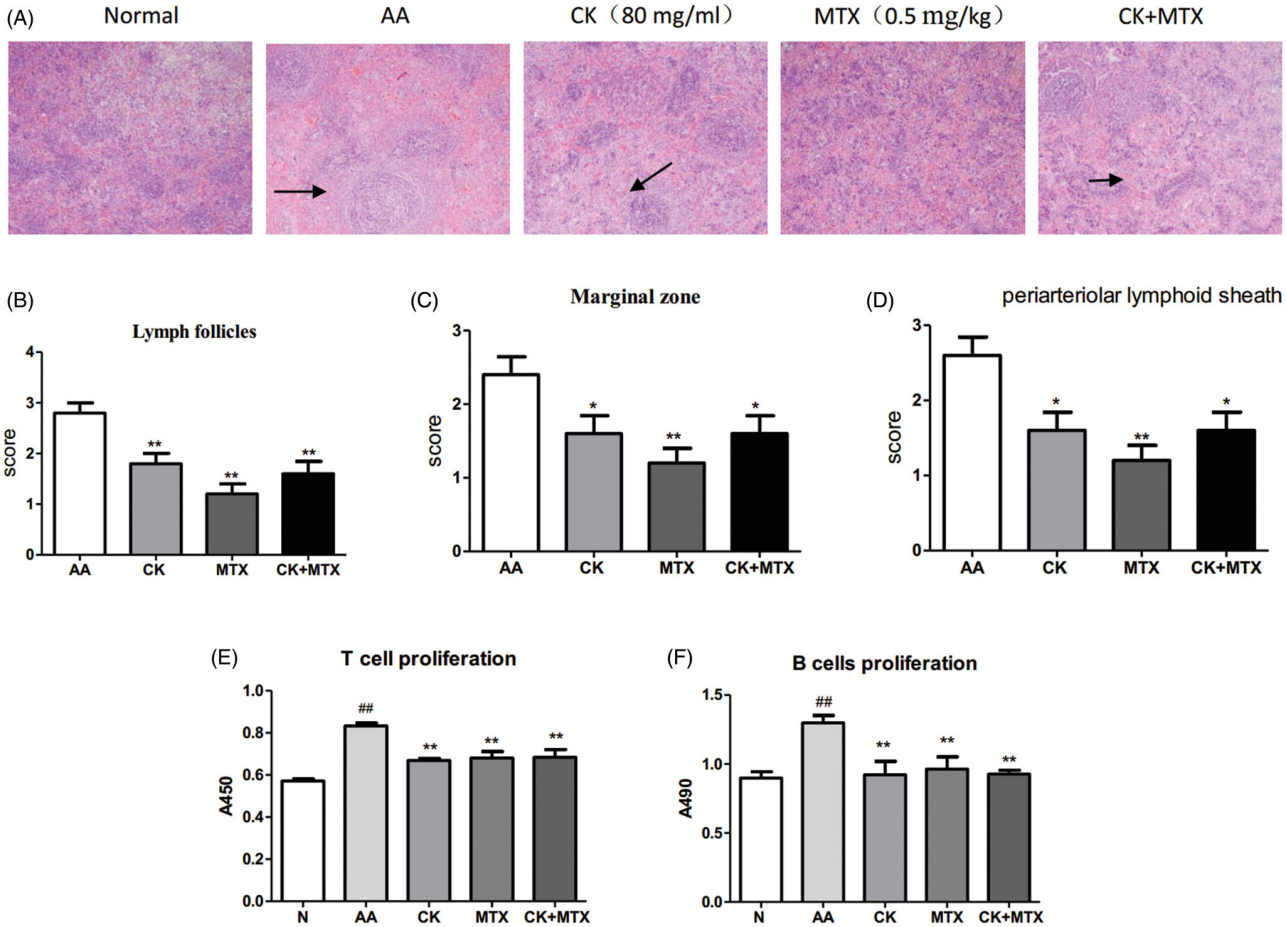
Combined effects ofCK and MTX on spleen histopathology and proliferation of T cells and B cells. (A) Photomicrographs of the rat spleen (original magnification × 100, haematoxylin and eosin stain), (B) Lymphoid follicles, (C) Marginal zone, (D) the periarteriolar lymphoid sheaths, (E) T cell proliferation, (F) B cell proliferation. Data are expressed as the mean ± SD, with 6 animals in each group, ^##^*p* < 0.01 vs. Normal; *p < 0.05; **p < 0.01 vs. AA.

In the spleen of AA rats, hyperplasia of white pulp was obvious, which evaluated an extensive activation, proliferation, and differentiation of T cells and B cells. Combination of CK and MTX significantly attenuated these changes (Figure 4(A–D)). In AA rats, ConA-induced T cell proliferative response and LPS-induced proliferation of B cell were significantly increased. Compared with AA model, combination of CK and MTX could significantly down-regulate the proliferation of T and B cells (Figure 4(E,F)).

**Figure 5.**
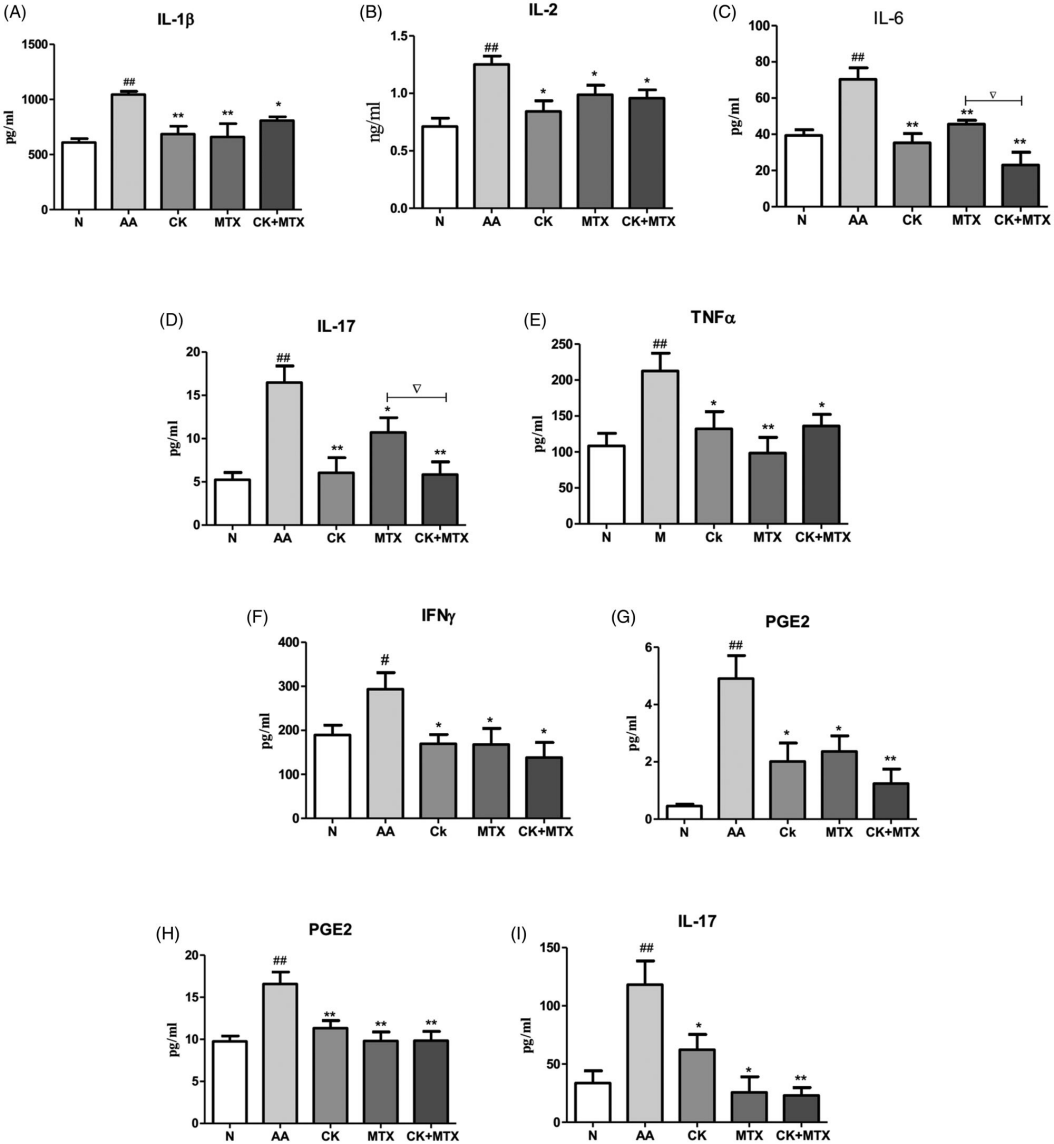
Combined effects of CK and MTX on pro-inflammatory cytokine in AA rats. pro-inflammatory cytokine in serum (A) IL-1β, (B) IL-2, (C) IL-6, (D) IL-17, (E) TNF-α, (F) IFN-γ, (G) PGE-2; (H) the secretion of PGE2 from macrophage; (I) the secretion of IL-17 from macrophage. Data are expressed as the mean ± SD, with 6 animals in each group. ^#^*p*＜0.05; ^##^*p*＜0.01 vs. Normal; *p＜0.05; **p＜0.01 vs. AA; ^▽^*p*＜0.05 vs. MTX.

Results showed that IL-1β, IL-2, IL-6, IL-17, TNF-α, IFN-γ, PGE2 in serum were markedly enhanced in AA rats, and combination of CK and MTX could reduce these cytokines (Figure 5(A–G)). Combination of CK and MTX was superior to MTX alone in reduction IL-6 and IL-17 in serum. Combination therapy showed decreased IL-6 to 25.1 ± 17.2 pg/mL compared with MTX group (44.9 ± 4.8 pg/mL), and decreased IL-17 to 5.8 ± 3.9 pg/mL compared with MTX group (10.7 ± 4.2 pg/mL) (Figure 5(C,D)). Compared with the normal group, PGE2 and IL-17 secreted by macrophages from AA rats were increased. Combination of CK and MTX could significantly suppressed the secretion of PGE2 and IL-17 from macrophages (Figure 5(H,I)).

## Discussion

Anaemia related to RA is a typical anaemia caused by chronic disease. Although this anaemia is usually moderate, low haemoglobin is closely related to the disease activity of RA, and can be used to predict the joint injury in early stage of RA (Möller et al. 2018). As the common extra-articular symptom of RA, anaemia is affected by many factors. For example, cytokines affect erythrocyte precursors differentiation, and anti-arthritic drugs affect haematopoietic function. Drugs used to treat RA in clinic include disease-modifying anti-rheumatic drugs (DMARDs), steroidal anti-inflammatory drugs, non-steroidal anti-inflammatory drugs (NSAIDs), biological agents and natural drugs (Laev and Salakhutdinov 2015). As a classical DMARD drug, low-dose MTX reduces or prevents joint damage in RA patients, but it works slowly, and long-term use of MTX can cause adverse reactions, which mainly including hepatotoxicity and hematological problems (Kim et al. 2018; Lin et al. 2019; Satou et al. 2019; Tabata et al. 2019). Hepatic damage and gastrointestinal problems caused by MTX alter the available iron stores, and then lead to an anaemic condition. In addition, the antifolate effect of MTX contributes to the occurrence of anaemia in MTX-treated RA patients. In present study, the remarkably decreased haemoglobin concentration in peripheral blood of AA rats indicated anaemia in AA rats, which was similar to RA in humans. The reticulocytes in peripheral blood significantly elevated after administration of MTX in AA rats, which suggested an anaemic condition in MTX-treated AA rats.

The best therapy for anaemia in RA patients is to control systemic disease by DMARDs or biological drugs. Combination of infliximab or tocilizumab and MTX promoted haemoglobin concentration in RA patients with anaemia (Doyle et al. 2009; Sun et al. 2018). Inconsistent with these cases, a RA patient developed a refractory anaemia after a classic therapy of infliximab and MTX for many years (D'Alessandro et al. 2011). Most importantly, long-term use of biological drugs could induce another serious adverse reaction, such as infection and tumour. Although combination of MTX and folic acid could ameliorate MTX-induced anaemia, the efficacy of MTX in RA could be minimized by this combination therapy. It is meaningful to search for a rational combination therapy in the treatment of RA, which can achieve better efficacy and alleviate anaemia at the same time.

In this study, arthritis signs were observed to evaluated the therapeutic effect of CK, MTX, and combination therapy. Histopathology of spleen, proliferation of T cells and B cells, and pro-inflammatory cytokines were assayed to evaluated the effect of drugs on immune function of AA rats. Histopathology of joint, proliferation of synoviocytes, and secretion of RANKL and OPG from synoviocytes were assayed to evaluated the effect of drugs on joint erosion and synovium inflammation in AA rats. CK, MTX, and combination therapy all could relieve arthritis signs and down-regulate immune function and suppress joint from erosion and synovium inflammation in AA rats. Combination of CK and MTX was superior to CK alone or MTX alone in reduction arthritis signs of AA rats, and in reduction of pro-inflammatory cytokines IL-6 and IL-17. Though the pathogenesis of anaemia in RA is still not completely understood, it is generally regarded to be connected with various increased cytokines. The increased cytokines (TNF-α, IFN-γ, IL-1 and IL-6) not only directly promote the development of RA, but also inhibit differentiation and proliferation of erythroid progenitor cells, result in the blunt response of erythropoietin to anaemia. Iron utilisation barrier induced by IL-6 plays an important role in RA-related anaemia. Treatment that blocks IL-6 is effective in RA with anaemia, such as the IL-6 receptor blocker tocilizumab. In trials of tocilizumab in RA with anaemia, haemoglobin levels significantly increased after the treatment (Genovese et al. 2008). In this study, combination of CK and MTX alleviated anaemia in AA rats, which might be related to the significantly decreased IL-6 after combination therapy. In addition, IL-6, a multipotent pro-inflammatory cytokine, plays a critical role in the systemic inflammatory response of RA. In RA patients, IL-6 in serum and synovial fluid was related with disease activity, number of affected joints, morning stiffness and X-ray changes of joints (Cronstein 2007). In RA, IL-6 induced the expression of RANKL and osteoclast differentiation, and promoted formation of a synovial pannus and destruction of joint (Feng et al. 2016). IL-17 triggered the inflammatory changes in the synovium at early stage, and maintained synovitis and bone erosion at late chronic stage of RA (Alves et al. 2016; Robert and Miossec 2018). In addition, IL-17 promoted inflammatory cytokine secretion, pannus formation and osteoclastogenesis in RA. In this study, the more efficiency of combination therapy in improving signs of arthritis in AA rats may be due to remarkably reduced IL-6 and IL-17 levels.

Ginseng is commonly used as traditional Chinese medicine to promote haematopoietic effects. CK comes from the degradation of products of natural ginsenosides, such as Rb1 in human intestinal tract. It has been reported that panaxadiol saponins including ginsenoside Rb1 remarkably stimulate haematopoietic function. Panaxadiol saponins components, in which the main ingredient is GRb1, have positive therapeutic effects on severe aplastic anaemia (Gao and Chong 2012; Zhang et al. 2017). In mice of immune-mediated aplastic anaemia, GRb1 remarkably improved haematopoietic function of bone marrow (Wen et al. 2016). Ginsenosides Rb significantly increased erythrocyte and haemoglobin in tumour mice, which suggested that ginsenosides Rb could improve anaemia (Zheng et al. 2019). It was reported that ginsenosides Rb1 increased peripheral blood haemoglobin and bone marrow stem cells, and these effects was related to the regulation of the proportions of T-cell subsets (Zhang et al. 2017; Zheng et al. 2019). CK is the main active product of Rb1, and the regulatory effect of CK on T cells activation has been proved in our previous studies (Chen et al. 2014, 2015), so the regulation of T-cells may contribute to the effect of CK in elimination of anaemia. Recently, it has been reported that CK could control apoptosis and promote cells to enter the normal cell cycle through Bcl-2/Bax and MEK/ERK, and exert protective effect on myelosuppression induced by cyclophosphamide in mice (Han et al. 2019). Based on these studies, we can speculate that the effect of combination of CK and MTX on anaemia in AA rats may be due to the protective effect of CK on myelosuppression and regulatory effect on T-cells.Steroidal anti-inflammatory drugs can be used in the treatment of RA and anaemia in RA. Glucocorticoid receptor (GR) is receptor of steroidal anti-inflammatory drugs, and plays a critical role in anti-inflammation and immunoregulation. But adverse reactions of steroidal anti-inflammatory drugs caused by long-term use limit its usage in clinic. It was reported that the mechanism of CK was related to activation of GR (Yang et al. 2008). Our previous studies also found that the anti-inflammatory and immunomodulatory effects of CK were related to the activation of GR (Wang et al. 2016). In addition, there was no serious adverse events of CK had been found in a preclinical long-term toxicity study and a phase I clinical study (Li et al. 2020; Chen et al. 2017). So may be CK can exert therapeutic effect of steroidal anti-inflammatory drugs without adverse reaction. It was reported that MTX *in vitro* induced greater expression of GR in PBMC from RA patients (Gatica et al. 2011). Although the mechanism of synergistic effect of CK combined with MTX in AA rats is still unclear. We can speculate that MTX increases the amount of GR, which can be activated by CK, thus producing synergistic effect in reduction of disease activity and elimination of anaemia in AA rats. Further studies should be done to verify the mechanism and the possibility in clinical usage.

## Conclusions

Combined CK with MTX achieved better efficacy and alleviated anaemia in AA rats. CK can be a candidate effective drug for combined treatment in RA patients with anaemia considering the better efficiency and the benefit on alleviation of anaemia. Our work supports a new combination therapy of MTX for RA patients.
